# NMR Structure of Hsp12, a Protein Induced by and Required for Dietary Restriction-Induced Lifespan Extension in Yeast

**DOI:** 10.1371/journal.pone.0041975

**Published:** 2012-07-27

**Authors:** Andrew P. Herbert, Michèle Riesen, Leanne Bloxam, Effie Kosmidou, Brian M. Wareing, James R. Johnson, Marie M. Phelan, Stephen R. Pennington, Lu-Yun Lian, Alan Morgan

**Affiliations:** 1 Department of Cellular and Molecular Physiology, Institute of Translational Medicine, University of Liverpool, Liverpool, United Kingdom; 2 NMR Centre for Structural Biology, Institute of Integrative Biology, University of Liverpool, Liverpool, United Kingdom; 3 University College Dublin Conway Institute of Biomedical and Biomolecular Research, University College Dublin, Dublin, Ireland; University of Washington, United States of America

## Abstract

Dietary restriction (DR) extends lifespan in yeast, worms, flies and mammals, suggesting that it may act via conserved processes. However, the downstream mechanisms by which DR increases lifespan remain unclear. We used a gel based proteomic strategy to identify proteins whose expression was induced by DR in yeast and thus may correlate with longevity. One protein up-regulated by DR was Hsp12, a small heat shock protein induced by various manipulations known to retard ageing. Lifespan extension by growth on 0.5% glucose (DR) was abolished in an *hsp12Δ* strain, indicating that Hsp12 is essential for the longevity effect of DR. In contrast, deletion of *HSP12* had no effect on growth under DR conditions or a variety of environmental stresses, indicating that the effect of Hsp12 on lifespan is not due to increased general stress resistance. Unlike other small heat shock proteins, recombinant Hsp12 displayed negligible *in vitro* molecular chaperone activity, suggesting that its cellular function does not involve preventing protein aggregation. NMR analysis indicated that Hsp12 is monomeric and intrinsically unfolded in solution, but switches to a 4-helical conformation upon binding to membrane-mimetic SDS micelles. The structure of micelle-bound Hsp12 reported here is consistent with its recently proposed function as a membrane-stabilising ‘lipid chaperone’. Taken together, our data suggest that DR-induced Hsp12 expression contributes to lifespan extension, possibly via membrane alterations.

## Introduction

It is commonly accepted that similar fundamental cellular processes modulate ageing in most eukaryotes [1]. Evidence to support this idea comes from studies of dietary restriction (DR), i.e. underfeeding without malnutrition [2]. DR extends lifespan in most model organisms, including yeast, worms, flies, and mammals [3], [4], suggesting that it may act via conserved longevity mechanisms. Studies using the budding yeast, *Saccharomyces cerevisiae*, have been at the forefront of recent efforts to understand the molecular mechanism of action of DR. Reducing the concentration of glucose in yeast growth media from the standard 2% to 0.5% or below increases both replicative and chronological lifespan in multiple genetic backgrounds and has been suggested to be a model of DR [5], [6], [7], [8].

Although the effect of glucose limitation in extending yeast replicative lifespan is not disputed, its mechanism of action remains the subject of considerable debate. One popular model postulates that DR increases lifespan by activating the NAD-dependent histone deacetylase, Sir2, or its homologues, resulting in increased ribosomal DNA (rDNA) silencing and a consequent reduction in rDNA recombination [6], [9], [10], [11]. An alternative theory is that DR extends lifespan in a Sir2-independent manner by inhibition of the Tor and Sch9 kinase signalling pathways [12], [13]. In this latter model, the downstream molecular mechanisms effecting longevity are not entirely clear, but may include reduced ribosomal protein biogenesis [13], [14] as well as reduced rDNA recombination [15].

To shed light on the mechanisms by which DR extends yeast lifespan, we set out to use an unbiased strategy to identify proteins whose expression is induced by DR and thus correlate with longevity. Hsp12, a small heat shock protein whose cellular functions are unclear, was identified by this approach and was found to be essential for lifespan extension by DR. At the molecular level, Hsp12 was found to be unlike other small heat shock proteins, in that it is monomeric and intrinsically unfolded in solution and has negligible *in vitro* chaperone activity. Upon binding to membrane-mimetic SDS micelles, Hsp12 switches to a 4-helical conformation. The NMR structure of micelle-bound Hsp12 determined here is consistent with its recently proposed function as a membrane-binding ‘lipid chaperone’ [16], which may in turn explain Hsp12’s ability to modulate various phenotypes and cellular functions.

## Results

**Figure 1:**
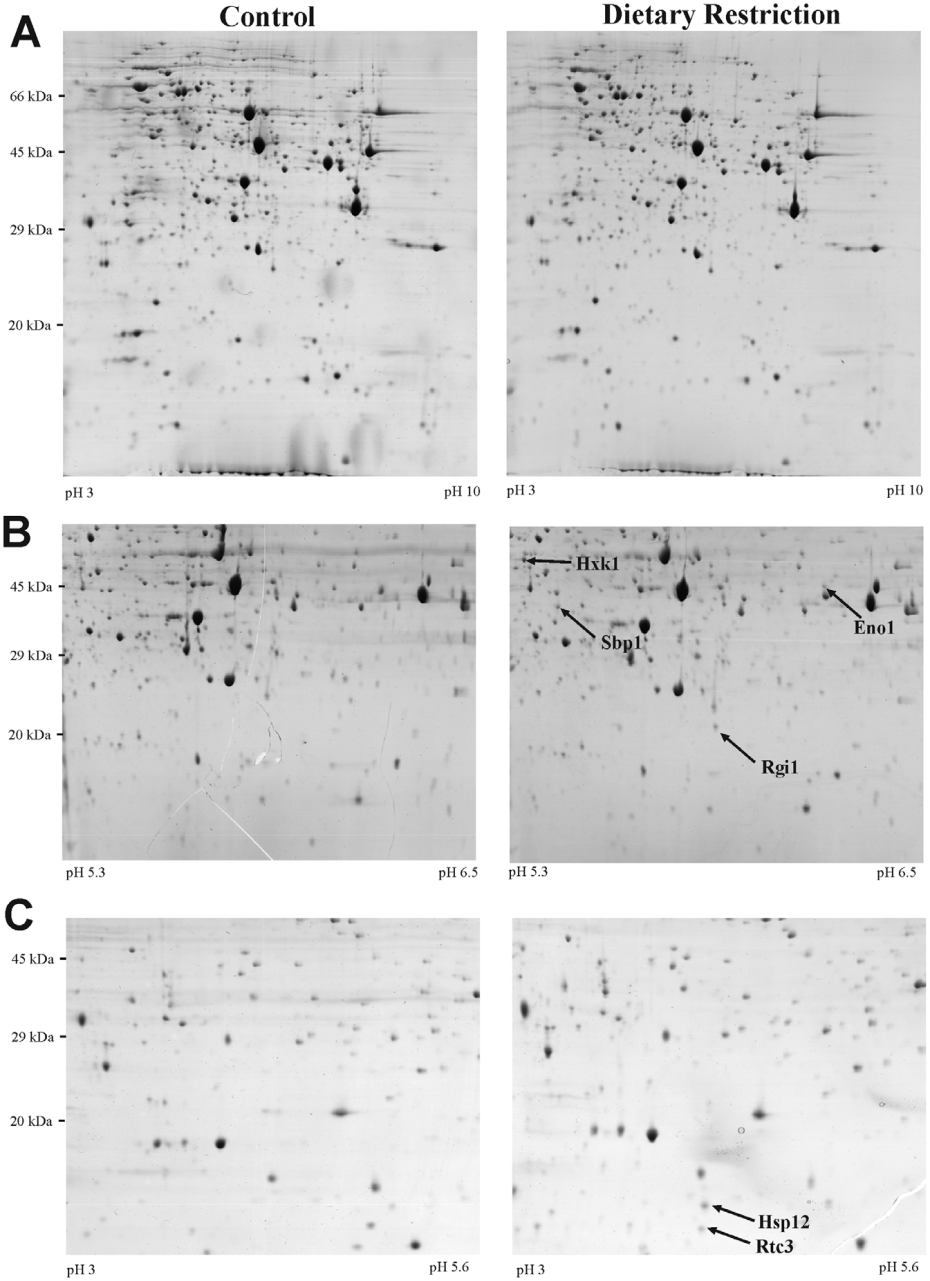
DR induces expression of a relatively small number of proteins. Wild type BY4741 yeast cells were grown in standard (2% glucose) and DR (0.5% glucose) conditions before lysis and separation of proteins by 2-D electrophoresis. Wide-range (pH 3–10) gels revealed no obvious reproducible differences in protein expression, as illustrated by representative gels shown in panel (A). Narrow pH range gels (pH 3–5.6 and 5.3–6.5) revealed changes in protein spots, which were identified by mass spectrometry. Selected identified proteins are indicated by arrows in panels (B) and (C).

To identify proteins that are induced by DR, we analysed extracts from BY4741 yeast cells grown under standard (2% glucose) or DR (0.5% glucose) conditions by 2-D gel electrophoresis. Initial experiments using wide range (pH 3–10) gels revealed no obvious reproducible changes in protein spot abundance, indicating that DR does not cause gross proteomic alterations (Fig. 1A). However, using pH 5.3–6.5 and pH 3–5.6 zoom gels, we could resolve several proteins that were barely detectable in control conditions, yet clearly induced in DR conditions (Fig. 1B, C). Mass spectrometry was used to identify these differentially expressed proteins as Eno1, Hxk1, Hsp12, Rtc3, Rgi1, Sbp1 and Yef3 (Table S1).

To help pinpoint DR-induced proteins that play a causal role in mediating lifespan extension, as opposed to those whose expression patterns are merely coincidental, we then analysed proteins that were induced by high osmolarity (Fig. S1). Our rationale was based on the observation that lifespan extension by DR and high osmolarity act via a common downstream pathway [17]; and hence the crucial effector proteins are likely to be common to both interventions. Proteins induced by high osmolarity comprised Ctt1, Eno1, Fba1, Hsp12, Hsp26, Hsp31, Lys9, Rtc3, Rgi1 and Oye2 (Table S1). To validate these protein expression changes, we prepared extracts from yeast containing chromosomally-tagged GFP fusion constructs [18] and performed western blots using a GFP antibody. Examples of proteins selectively induced by DR (GFP-Hxk1) or high osmolarity (GFP-Ctt1) are shown in Fig. S1B. Of the proteins identified as being induced by both interventions, specific bands of the predicted size could not be reproducibly detected for GFP-Rtc3 or –Rgi1; whereas GFP-Eno1 expression was not altered by DR. However, GFP-Hsp12 was confirmed to be induced by both DR and high osmolarity (Fig. S1B). To rule out any artefactual effect of the GFP tag, we raised an antiserum against the N-terminus of Hsp12 and used this in western blots of wild type cells. This revealed a band of the expected size (∼12 kDa), which was increased in intensity upon growth in DR and high osmolarity conditions and which was not present in an isogenic *hsp12* deletion strain (Fig. S1C), thus confirming the increased expression of Hsp12 under conditions of enhanced longevity.

**Figure 2:**
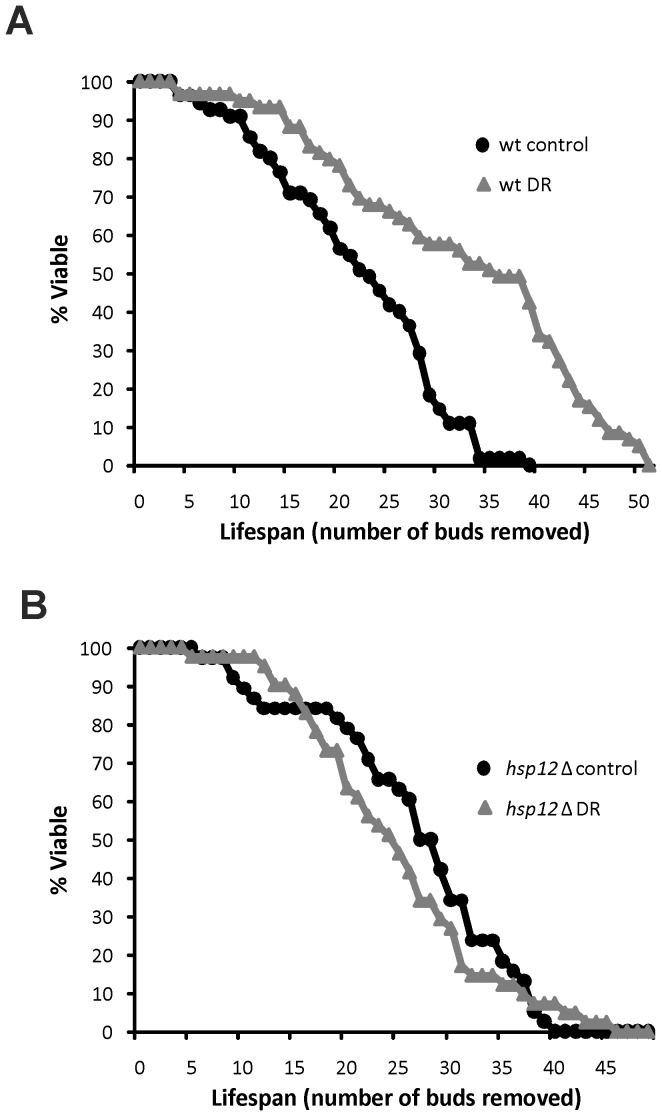
*HSP12* is essential for lifespan extension by DR. (A) The replicative lifespan of the wild type BY4741 strain grown under standard (2% glucose) and DR (0.5% glucose) conditions was determined manually on YPD plates by micromanipulation. Mean and median lifespan were 21 and 22, respectively, (n = 55) for 2% glucose; and 31 and 35, respectively, (n = 59) for 0.5% glucose. DR produced a significant increase in lifespan in wild type cells (log-rank test, *P*<0.001) (B) Mean and median lifespan of the isogenic *hsp12*Δ strain was determined as above and found to be 26 and 28, respectively, (n = 39) for 2% glucose; and 25 and 24, respectively, (n = 42) for 0.5% glucose. DR did not increase lifespan in *hsp12*Δ cells (log-rank test, *P*>0.5).

To determine if Hsp12 is causally linked to DR-induced lifespan extension, we performed replicative lifespan analysis. This was done by determining the number of daughter cells removed by micromanipulation from individual virgin mother cells [19]. Wild type BY4741 cells exhibited a mean lifespan of 21 (95% CI: 19–23) on standard (2% glucose) media, which was significantly increased to 31 (95% CI: 28–35) under DR (0.5% glucose) conditions (Fig. 2A). Deletion of *HSP12* did not reduce longevity under standard conditions, but rather resulted in a small increase in mean lifespan to 26 (95% CI: 23–29). Strikingly, however, the ability of DR to increase longevity was abolished in the *hsp12*Δ strain, which exhibited a mean lifespan of 25 (95% CI: 22–28) under DR conditions (Fig. 2B). These data suggest that the impact of Hsp12 on cellular ageing is complex: the low-level expression observed in standard media has a small negative effect on lifespan, whereas high Hsp12 levels induced by DR are essential for the increase in longevity caused by this intervention.

Stress resistance correlates positively with lifespan in various model organisms and DR may represent a mild stress that extends lifespan via a hormesis-like mechanism [20]. We therefore investigated if deletion of *HSP12* reduced resistance to environmental stresses. For comparison, we also included *sir2* and *fob1* deletion strains in this analysis, as deletion of *SIR2* and *FOB1* is known to decrease and increase replicative lifespan respectively 21,22. There was no detectable difference between the ability of BY4741 wild type and the deletion mutants to grow under a wide variety of stress conditions, including DR and other stresses that increase Hsp12 expression (Fig. S2). We therefore conclude that Hsp12 does not contribute to general stress resistance.

Various small heat shock proteins have been shown to be ‘holdase’ molecular chaperones that bind to denaturing proteins and prevent their aggregation. To determine if Hsp12 had such activity, we investigated the ability of recombinant purified Hsp12 to prevent aggregation of the model substrate, insulin, using the method of Haslbeck *et al.* [23]. Addition of DTT reduces the disulphide bonds between the A and B chains of insulin, causing aggregation; whereas in the absence of DTT, insulin remains stable (Fig. S3A). DTT-induced insulin aggregation was greatly reduced by recombinant GST-fusion proteins of the known chaperones, yeast Hsp26 [23] and mammalian cysteine string protein (CSP) [24]; but not by CaBP1s, used as a control for a protein of similar size to Hsp12 with no known or predicted chaperone functions (Fig. S3B). However, GST-Hsp12 was similar to GST-CaBP1s in terms of ability to prevent insulin aggregation. The differences in chaperone activity for GST-Hsp12 and GST-Hsp26 were then assessed in a dose-dependent manner. This revealed that GST-Hsp26 has approximately 100-fold higher anti-aggregation activity than GST-Hsp12 (Fig. S3C), indicating that Hsp12 has very low, if any, intrinsic chaperone activity.

**Figure 3:**
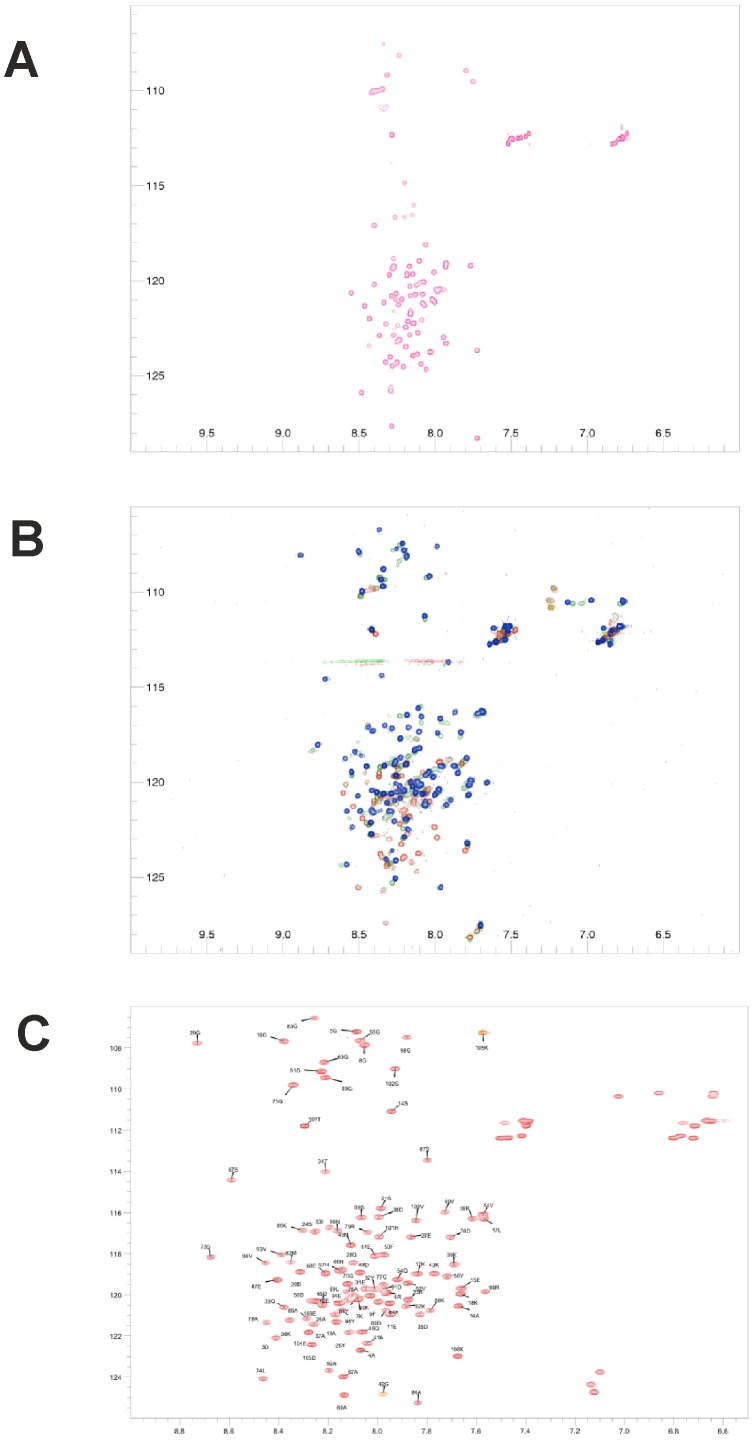
Hsp12 is unstructured in solution, but folds in the presence of SDS. (A) ^1^H-^15^N HSQC spectrum of Hsp12 in aqueous solution at 298 K. The spectrum shows only sharp peaks with random coil shifts indicating the absence of any structured regions. (B) ^1^H-^15^N HSQC spectrum of Hsp12 at 303 K in the presence of increasing concentrations of SDS (0, 1, 2, 5, 8 mM Red -> Blue). SDS causes a considerable increase in the amount of chemical shift dispersion implying increased levels of folded material/regions. (C) Assigned ^1^H-^15^N HSQC spectrum of Hsp12 at 318 K in the presence of 100 mM SDS.

In addition to possessing anti-aggregation properties, small heat shock proteins are often large homo-oligomeric assemblies of folded subunits. To further investigate the possible function of Hsp12, we determined its solution structure using NMR. Recombinant Hsp12 expressed in *E. coli* was monomeric. The ^15^N-^1^H HSQC spectrum showed poor resonance dispersion in the proton dimension, which suggested that Hsp12 is intrinsically disordered in aqueous buffer (Fig. 3A). Recently published circular dichroism studies have shown that Hsp12 gains significant helical content upon binding to lipid or SDS micelles [16], we therefore examined the effect of varying SDS concentrations. The ^15^N-^1^H HSQC spectra of Hsp12 showed a dose-dependent increase in dispersion in response to SDS, indicating that Hsp12 adopts a folded conformation upon micelle binding (Fig. 3B). Having determined the optimal SDS concentration for NMR, we then characterised the temperature-dependence of Hsp12 in the presence (Fig. S4A) and absence (Fig. S4B) of SDS. This resulted in linear resonance dispersion until 45°C, above which some resonances deviated from a straight line in the presence of SDS, indicating heat-induced unfolding. These optimised conditions were then used to assign the residues of SDS-bound ^15^N/^13^C-labelled Hsp12 (Fig. 3C).

**Figure 4:**
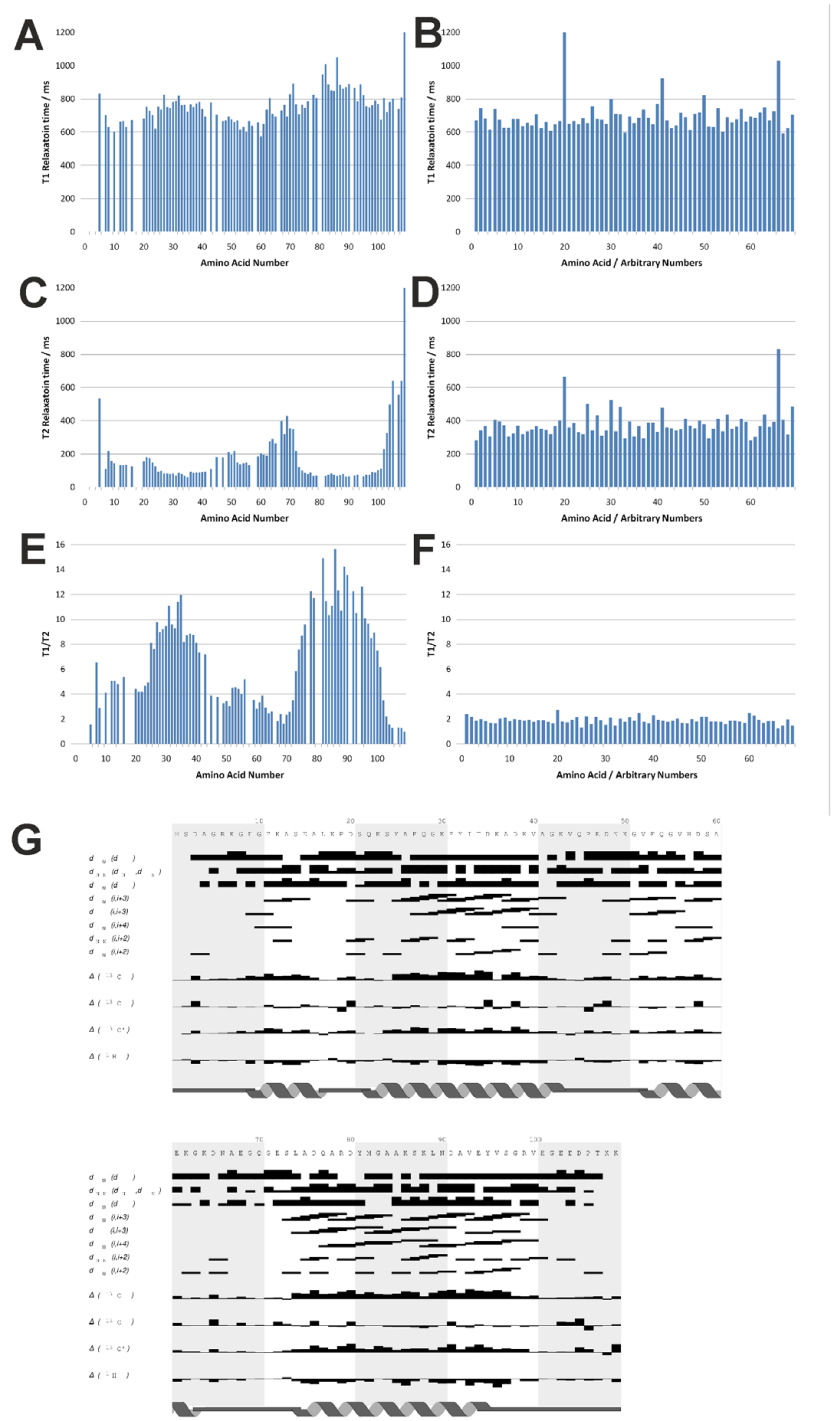
Backbone dynamics and chemical shift-based secondary structure of Hsp12. *T*_1_, *T*_2_ and *T*_1_/*T*_2_ relaxation values are shown for Hsp12 in the presence (A,C,E) and absence (B,D,F) of 100 mM SDS at 318 K. *T*_1_ and *T*_2_ relaxation times for micelle-bound (A,C) Hsp12 show significant variation; contrasting with the similar relaxation values observed for free Hsp12 (B,D). Micelle-bound Hsp12 (E) shows grouped variations in the *T*_1_/*T*_2_ values ranging from approximately 1.5 to 14, indicating a wide range of mobility and a clear differentiation of secondary structure elements; whereas the free form (F) shows consistent values of around 2, indicating a completely unstructured protein. (G) The assigned chemical shifts at 318 K in 100 mM SDS expressed as deviation from random coil are shown aligned with the primary sequence and the positions of the α-helices.

Analysis of the backbone dynamics of Hsp12 in the presence of SDS revealed relatively long *T*_1_ relaxation values compared to *T*_2_ (Fig. 4 A,C,E), suggesting restricted mobility in the majority of the polypeptide. In contrast, *T*_1_ and *T*_2_ values were similar in the absence of SDS (Fig. 4 B,D,F), suggesting that the protein is highly dynamic in solution, but is structured on micelles. Consistent with this, analysis of the assigned chemical shifts in Hsp12 using CSI [25] suggested that micelle binding induces the formation of four α-helices (Fig. 4G). These α-helices cover the majority of the polypeptide and comprise residues F9-A16 (Helix I), Q22-A41 (Helix II), V52-G63 (Helix III) and L74-E94 (Helix IV). Helix III is not as stable as the other four helices, as revealed by the lower number of d_αNi,i+3_, d_αβi,i+3_ connectivities for this helix and more variation in its length compared with the other three helices together with a high RMSD value of 0.465 (Table S2). The experimentally-determined structural data correspond well with prediction using the AGADIR programme [26], which shows that the region between 52–63 has a lower helical propensity compared with the other three helical regions. Extensive analysis of residual dipolar couplings using stretched acrylamide gels revealed no evidence of long-range interactions between the individual helices, indicating that Hsp12 does not form a stably-folded structure.

**Figure 5:**
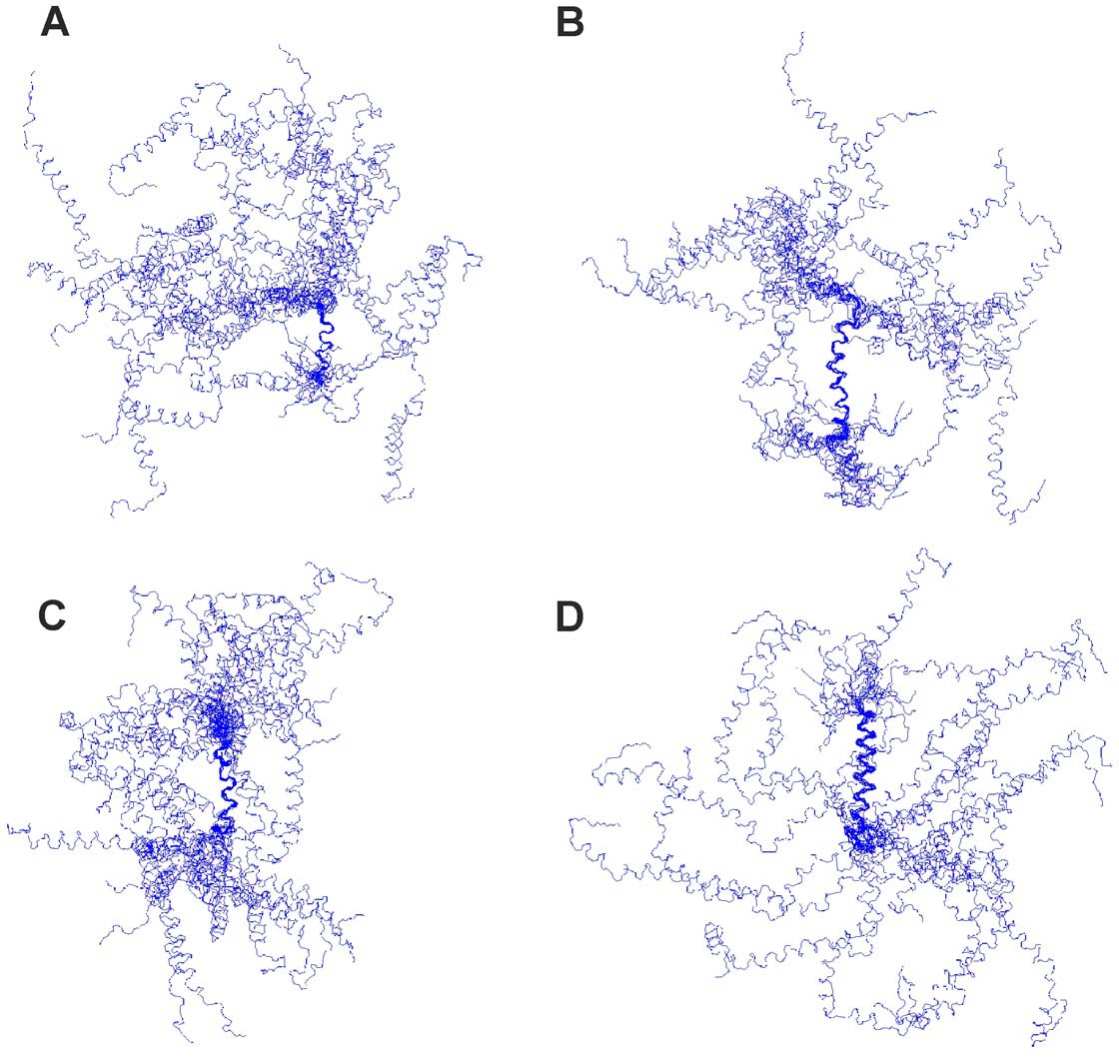
Ensemble of structures calculated for micelle-bound Hsp12 overlaid on each of the four helices. Ensemble of twenty structures overlaid on helices I (A), II (B), III (C) and IV (D). No long-range interactions were detected and so the helices appear free to move independently with no overall fold being evident.

**Figure 6:**
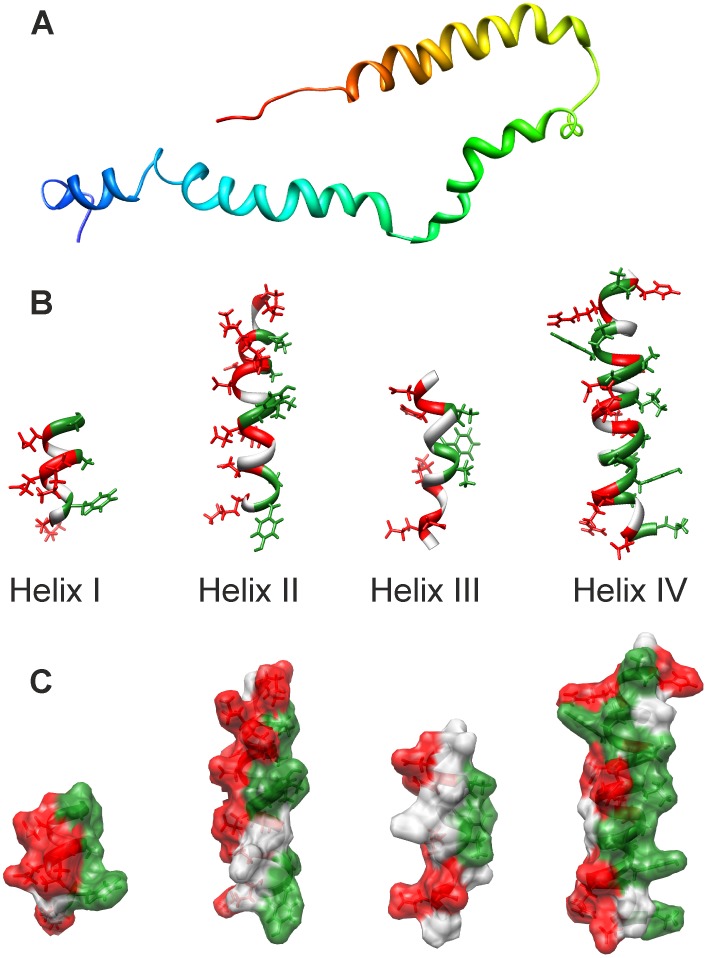
Helical properties of micelle-bound Hsp12. (A) The four α-helices are represented as ribbons and colour coded from the N-terminus (blue) to the C-terminus (red) in a representative structure. (B,C) Analysis of charge distribution with hydrophobic residues labelled green and charged residues labelled red in both ribbon (B) and surface (C) representation, illustrating the amphipathic nature of Hsp12. Structures were generated using Chimera.

We generated a model of the tertiary structure of Hsp12 using CYANA. The ensemble presented (Fig. 5 and Fig. S5) highlights the flexibility of the α-helices relative to one another. The four α-helices can be more clearly identified in the representative model in Fig. 6A, with the 4^th^ and most C-terminal helix represented in yellow/red. Analysis of the charge distribution reveals each α-helix to be broadly amphipathic, with hydrophobic (green) residues lying on one face and charged (red) residues on the opposite face (Fig. 6 B,C). In addition, the residues flanking each α-helix also tend to be charged. This suggests that hydrophobic residues of Hsp12 insert into the lipidic component of membranes, while the charged (mainly positive) residues interact with negatively charged head groups and project away from the membrane. A Ramachandran plot of the data is presented in Fig. S6. Overall, the NMR data indicate that Hsp12 is intrinsically unstructured in aqueous solution, but switches to a dynamic 4-helical conformation upon membrane binding.

## Discussion

The mechanisms by which DR extends yeast replicative lifespan remain unclear and controversial. The data presented here show that DR (via growth in 0.5% glucose) does not induce gross changes in overall protein abundance, but does result in up-regulation of a limited number of proteins. This is consistent with earlier global microarray analysis that revealed changes in mRNA expression for only 133 genes (around 2% of total ORFs) in response to growth in 0.5% glucose [9]. Some of the proteins we found to be upregulated by DR (e.g. Rgi1, Hxk1) are associated with metabolic adaptation from fermentation to respiratory growth; whereas the reason for up-regulation of other proteins (e.g. Hsp12, Rtc3) is less obvious. Both Hsp12 and Rtc3 are also induced by high osmolarity (Fig. S1), which is thought to extend lifespan via the same downstream mechanisms as DR [17], consistent with the idea that DR is a chronic mild stress that increases longevity via hormesis. Indeed, *HSP12* mRNA levels are increased in response to diverse environmental stresses, including heat-, osmotic- and oxidative stress [27], [28]. *HSP12* is also among the top 10 up-regulated genes in response to non-environmental, genetically-mediated impairment of nuclear proteostasis [29] and telomere capping [30].

Despite the strong induction of *HSP12* gene expression in response to diverse stresses, deletion of *HSP12* does not generally affect sensitivity to these stresses. Significantly, however, we found that lifespan extension by growth on 0.5% glucose was abolished in an *hsp12Δ* strain, indicating that Hsp12 is essential for the longevity effect of DR. What mechanism(s) could explain this action of Hsp12 on lifespan? One tempting explanation is that, like other small heat shock proteins, Hsp12 prevents the aggregation of a variety of cellular proteins during DR-induced stress. However, we found that Hsp12 has negligible anti-aggregation properties *in vitro*, in contrast to well established ‘holdase’ co-chaperones such as Hsp26. It therefore seems more likely that Hsp12’s recently discovered function as a membrane stabilising ‘lipid chaperone’ [16] underlies its effect on lifespan. Indeed, Ssd1 has been shown to increase replicative lifespan in a Sir2-independent manner by increasing plasma membrane stability [31]. The transition from unfolded Hsp12 in solution to a dynamic 4-helical lipid-bound conformation observed here may similarly contribute to membrane stability under DR conditions, which in turn may be functionally relevant to increasing lifespan. However, it remains possible that unfolded Hsp12 in the nucleus and cytosol performs membrane-binding-independent functions that impact on replicative lifespan during DR.

The structure of Hsp12 bound to SDS micelles described here provides insight into how Hsp12 may interact with cellular membranes. This involves a switch from an intrinsically disordered conformation in solution to a structured, 4-helical lipid-bound conformation, as recently proposed [16]. Our extensive NMR analysis using stretched acrylamide gels failed to reveal interactions between the four α-helices of Hsp12, suggesting a dynamic structure whereby each α-helix is free to move independently within a 2-dimensional lipid bilayer. The lack of a stable folded tertiary structure could be due to the observed instability of helix 3. While our work was being prepared for publication, the NMR structure of micelle-bound Hsp12 was independently reported [32]. This shows a similar 4-helical conformation in the presence of SDS, although some differences are apparent in the secondary structural models of the individual helices (F9-K18, Y25-G42, Q54-S59 and D76-V100 versus F9-A16, Q22-A41, V52-G63 and L74-E94 in our study). We propose that the dynamic nature of Hsp12’s four α-helices we report here could be important for its membrane stabilising function [16], as this would enable simultaneous linkage of several distinct membrane sub-domains via a single protein tether.

In summary, we have shown that Hsp12 is induced by DR, is required for lifespan extension by DR, and have solved the structure of the protein in the free- and micelle-bound state. Although Hsp12 does not have obvious homologues outside of fungi, down-regulation of small heat shock proteins by RNAi also affects ageing in *C. elegans* [33], consistent with a conserved modulatory role for sHSPs in longevity. Hsp12 displays low-level homology to two mammalian proteins: ageing-associated protein 2 (also known as HIP/ST13; NCBI reference NP_003923) and α-synuclein (NCBI reference P37840). Intriguingly, α-synuclein is also an intrinsically disordered protein in solution that becomes α-helical upon binding to anionic phospholipids and SDS micelles [34]. It is conceivable that these shared properties may have functional implications, as it has been suggested that neuronal α-synuclein organises and stabilises lipids in synaptic vesicle membranes [35]. It is tempting to speculate that enhanced membrane stabilisation by functional homologues of Hsp12 may be relevant to DR-induced longevity in higher organisms.

## Materials and Methods

### Chemicals and Reagents

Materials for yeast culture were obtained from Sigma-Aldrich (Poole, UK) and Foremedium (Norwich, UK). PCR primers were supplied by Sigma Genosys (Havenhill, UK), genomic DNA isolation kits were from Invitrogen (Paisley, UK); and PCR enzymes/reagents were from Promega (Southampton, UK). Anti-GFP antibody was obtained from AbCam (Cambridge, UK) and custom-generated Hsp12 antiserum was supplied by Genosphere Biotechnologies (Paris, France). Materials for gel electrophoresis were obtained from GE Healthcare or BioRad. All other materials were obtained from Sigma-Aldrich.

### Yeast Strains

Deletion strains with the appropriate ORF replaced by the KanMX4 cassette in the BY4741 background (*MATa his3Δ1 leu2Δ0 met15 Δ0 ura3Δ0*) [36] were used for gel electrophoresis, lifespan analysis and stress assays and were obtained from Invitrogen (Paisley, UK). GFP-labelled strains in the BY4741 background were used to validate mass spectrometry identifications and were obtained from Invitrogen. All mutants were confirmed by PCR using gene-specific and KanMX primers.

### Yeast Cell Lysis and Protein Quantification

Single yeast colonies were grown in 5 ml of YP media containing the appropriate concentration of glucose overnight at 30°C. The next day, 4 µl of this starter culture was inoculated into 200 ml of YPD media YP media containing the appropriate concentration of glucose and incubated at 30°C C until an OD_600_ value of 0.6 was obtained. The glucose concentration remaining in the culture media fell by only 0.1% during this period (i.e., from 0.5% to 0.4%), as determined by glucose oxidase assays. At this point, the culture was centrifuged at 8000 g and washed 3 times with distilled water before freezing at −80°C. The equivalent of a 5-ml culture in a frozen pellet was resuspended in 150 µl lysis buffer (7 M urea, 2 M thiourea, 2% (w/v) CHAPS, 1% (w/v) DTT, 0.8% (w/v) Pharmalyte, 1 Roche protease inhibitor tablet per 10 ml), transferred to a 2 ml cryogenic vial and subjected to glass bead lysis in a mikrodismembrator for 10 min at 1800 rpm at 4°C. The resulting lysate was cleared by centrifugation at 13000 rpm for 10 min at 4°C. The protein concentration of each lysate was normalised by running samples together on a 1-D mini-gel, staining the gel with Coomassie blue and quantifying the total amount of protein in each track by densitometry. These values were then used to ensure that the same amounts of protein were loaded for each sample to be compared in subsequent 2-D gel electrophoresis or western blotting.

### 2-D Gel Electrophoresis and Protein Identification

Yeast lysates were applied at equal protein concentrations to 18 cm IPG strips (GE Healthcare) and allowed to rehydrate for a minimum of 10 hours. The IPG strips were then run on an IEF Multiphor II electrophoresis unit (GE Healthcare) at 20°C for 1 min at 500 V, then for 7 hours at 3500 V. Following this, IPG strips were overlayed on SDS-PAGE gels, sealed using warm agarose, and run using an Ettan Dalt II system (GE Healthcare) at 2.5 W per gel for 30 min and then 20 W per gel at 25°C until the dye front had reached the bottom of the gel. Immediately after SDS-PAGE, the gels were fixed in a solution of 40% v/v MeOH/7% (v/v) acetic acid for a minimum of 2 hours and then stained with colloidal Coomassie blue stain (GE Healthcare). Stained gels were imaged with a GS-710 Imaging Densitometer (BioRad) and protein spot changes analysed by eye and using PD Quest software (BioRad). Protein spots of interest were excised from gels, dried under vacuum, then re-hydrated in 25 mM NH_4_CO_3_ containing 5 ng/µl trypsin overnight at 37°C. Tryptic peptides were resuspended 1∶1 in matrix solution (10 mg/ml HCCA in 50% (v/v) ethanol, 50% (v/v) acetonitrile, 0.001% (v/v) trifluoroacetic acid) and 1 µl of this mixture (25% of the total digest) was spiked with 50 fmol of ACTH peptide, loaded onto a MALDI target (Waters/Micromass Massprep workstation) and analysed on a MALDI-TOF mass spectrometer (Waters/Micromass M@LDI). Tryptic peptides were identified by peptide mass fingerprint matching using MASCOT (UniProt release 2.6) allowing for 1 missed cleavage with a mass accuracy of 0.25 Da.

### Western Blotting

Yeast lysates prepared as above were run on 1-D SDS-PAGE gels and transferred to nitrocellulose. Blots were probed with either anti-GFP antibody (AbCam, Cambridge, UK) or with custom-generated antiserum (Genosphere Biotechnologies, Paris, France) raised against a synthetic peptide corresponding to the N-terminal 14 residues of Hsp12 with an additional cysteine for conjugation (sequence: MSDAGRKGFGEKASC). Blots were visualised by enhanced chemiluminescence and imaged with a BioRad ChemiDoc XRS imager (BioRad).

### Lifespan Analysis

This was performed as described previously [19]. Briefly, strains were grown at 30°C until they reached an OD_600_ of 0.6–1.0. One microlitre of culture was streaked onto plates and left at 30°C for 1–2 h. After this time, cell doublets were moved to uninhabited regions of the plate. When these budded again, (newly formed) virgin yeast cells were removed by micromanipulation to a new location. All future buds produced by these daughter cells were micromanipulated away and catalogued. The plates were incubated at 30°C during working hours, and moved to 4°C overnight. Lifespan was defined as number of daughter cells removed from the mother cell. All lifespans were observed at least twice. Statistical significance was assessed using the log rank test and deemed significant at *P*<0.05.

### Stress Assays

Single colonies were grown overnight in 5 ml liquid YP media containing 2% (w/v) glucose. The next morning, cultures were diluted to OD_600_ = 1 in sterile H_2_O and then serially diluted ten-fold five times in sterile H_2_O. The serial dilutions were then spotted with a replica plater onto YP media containing the indicated stressors. Plates were incubated at 30°C for 2 to 4 days and then imaged with a BioRad ChemiDoc XRS imager (BioRad).

### Recombinant Proteins

GST-Hsp12 and GST-Hsp26 were constructed using the Invitrogen Gateway cloning protocol. Primers for amplifying Hsp12 and 26 from yeast genomic DNA were designed from the ORF sequences of the required genes fused with the corresponding attB1 and attB2 primer sequences. The destination vector used for N-terminal GST-fusion, pG-GEX6p-B1, was made in-house. Recombinant GST-fusion proteins were expressed in BL21 (DE3) *E. coli*, lysed using a One Shot cell disrupter (Constant Systems, Daventry, UK) and purified using glutathione-Sepharose as previously described [37].

For NMR studies, the ORF of Hsp12 was codon optimised for *E. coli* expression and then synthesised *de novo* by GeneArt (Invitrogen). The resulting construct was then cloned into the pE-SumoProKan vector (Invitrogen) and expressed in BL21 (DE3) *E. coli* in M9 medium containing 1 g/L ^15^NH_4_Cl as the sole nitrogen source, with either 4 g/L ^12^C-glucose or ^13^C-glucose as the sole carbon source, to produce, respectively, [U-^15^N]- or [U-^15^N,^13^C]-Hsp12. Recombinant His-SUMO-tagged Hsp12 was purified using HisTrap immobilised metal affinity columns (GE Healthcare) via imidazole elution. The SUMO-specific protease, ULP1, was used to cleave Hsp12 (with no vector-derived residues) from the His-SUMO tag, which was removed by passage through a second immobilised metal affinity column. The resulting Hsp12 protein was then dialysed against 10 mM KHPO_3_, 40 mM NaCl, pH 6.5 and concentrated prior to use in NMR.

### Aggregation Assay

This was based on the method of Haslbeck *et al.* [23]. Insulin was added to a final concentration of 45 µM in 40 mM HEPES-KOH, pH 7.5 in a 96-well plate in the presence or absence of recombinant proteins. Aggregation was induced by the addition of 1.5 µl of 1 M DTT in a final volume of 100 µl, or by adding 1.5 µl of water as a no-aggregation control. Measurements were taken in an Emax microplate reader (Molecular Devices, Sunnyvale, USA) at 405 nm every 10 min for 3 hours, then one final reading was obtained the next day after agitating the plate to disperse any large aggregates. The anti-aggregation efficiency of each recombinant protein was calculated as follows. For each individual assay, the highest absorbance reading for the aggregation control (insulin + DTT) was assumed to reflect total aggregation of insulin (26 µg per well) and set as 100%. Anti-aggregation activity was then calculated by subtracting the aggregated material in each condition from 100%, converting to µg insulin and then dividing by the respective amount of each GST-tagged protein to determine the anti-aggregation properties per µg recombinant protein.

### NMR Methods

NMR samples were prepared in 10 mM PO_4_^3−^, 40 mM NaCl, 2 mM NaN_3_, pH 6.5 in 90% (v/v) H_2_O/10% (v/v) D_2_O or 100% D_2_O, at a final protein concentration of approximately 450 µM. Hsp12 samples in the presence of SDS were prepared using the same buffer with the addition of 100 mM SDS. Spectra in the absence and presence of SDS were acquired at, respectively, 303 K and 318 K, on Bruker Avance III 600 and 800 MHz spectrometers equipped with cryogenic triple resonance probes. All NMR spectra were processed with TopSpin (Bruker) and analysed using the CCPN Analysis package [38]. Sequence-specific backbone and side-chain resonance assignment of Hsp12 was made using 3-D HNCA, HN(CA)CB, HN(CO)CA, HNCO, CBCA(CO)NH, HBHANH, HBHA(CO)NH and HCCH-TOCSY experiments. Assignment of aromatic side-chain residues was made using 2-D [^1^H-^13^C] HSQC and homonuclear ^1^H NOESY and TOCSY spectra. Interproton distance restraints were obtained from NOEs derived from 3-D ^15^N- and ^13^C-edited NOESY-HSQC experiments. The secondary structure for the Hsp12 protein ensemble was determined using the STRIDE algorithm [39]. The basis for secondary structure is a combination of backbone torsion angle and location of hydrogen bonds. Furthermore, using the ensemble for secondary structure determination enabled the location of the helices to be identified based on twenty structures rather than one. RMSD values for the ensemble were determined by global rmsd fit using MOLMOL [40] which determines the average pairwise alignment RMSD for all pairs in the ensemble. IPAP ^1^H ^15^N RDC data was collected unaligned and aligned using the stretched gel method [41] on an 800 MHz spectrometer equipped with TCI cryoprobe. For each sample a 600 µl preparation of gel mix (4–8% acrylamide), containing a final concentration of 100–200 µM protein, was used and quadrupole splitting measured to estimate the degree of alignment. RDCs were collected on a 4.0% acrylamide gel of initial 6 mm diameter compressed into a 4.6 mm diameter tube with quadrupole splitting of 1.5 Hz containing 150 µM ^15^N Hsp12 and buffer conditions: 100 mM SDS, 10mM PO_4_^3−^, 40 mM NaCl, pH 4.5. Couplings were measured using CCPN analysis and a purpose-built Perl script, before direct incorporation into structure calculation using CYANA [42]. Coordinates have been deposited in the RCSB protein databank (PDB accession number: 4AXP) and the Biological Magnetic Resonance Bank (BMRB accession number: 18523).

## Supporting Information

Figure S1 **Hsp12 is induced by multiple lifespan-extending interventions.** (A) Wild type BY4741 yeast cells were grown in standard (2% glucose) and high osmolarity (20% glucose) conditions before lysis and separation of proteins by 2-D electrophoresis on narrow pH range gels (pH 3.5–6 and 5.3–6.5). Selected spot changes identified by mass spectrometry are indicated by arrows. (B) S288c yeast cells expressing chromosomally GFP-tagged fusions of selected proteins identified as being induced by DR or high osmolarity were grown in 0.5%, 2% and 20% glucose, separated by 1-D SDS-PAGE and western blotted with anti-GFP antiserum. (C) Wild type BY4741 yeast grown in 0.5%, 2% and 20% glucose (left panel); or wild type and isogenic *hsp12*Δ strains grown in 0.05%, 0.5% and 2% glucose (right panel) were separated by 1-D SDS-PAGE and western blotted with anti-Hsp12 antiserum.
(TIF)

Figure S2 ***HSP12*** **is not required for general stress resistance.** Overnight cultures of BY4741 wild type and deletion strains were serially diluted and then spotted with a replica plater onto YPD plates containing 2% glucose at 30°C unless indicated otherwise. Plates were incubated at 30°C for 2 to 4 days and then imaged in a BioRad Universal Hood II Imager (BioRad).
(TIF)

Figure S3 **Recombinant Hsp12 has negligible in vitro chaperone activity.** (A) 45 µM insulin was supplemented with 1.5 µl water (open squares) or 1 M DTT (black squares) and aggregation over time at room temperature was measured at A_405_ in a microplate reader. (B) Aggregation assays were performed as above in the presence or absence of the indicated GST-fusion proteins. Anti-aggregation activity is shown as the amount of insulin in µg which is prevented from aggregation by 1 µg of recombinant protein. Data shown are pooled from multiple experiments (n = 7 for GST-Hsp12; n = 8 for GST-Hsp26; n = 4 for GST-CSP; n = 4 for GST-CaBP1s). The difference between GST-Hsp12 and GST-CaBP1s was deemed significant at *P*<0.05 using a Student’s *t*-test. (C) Dose-response curves of recombinant GST-Hsp12 and GST-Hsp26. GST-Hsp26 greatly reduces insulin aggregation, whereas GST-Hsp12 has mimimal effect.
(TIF)

Figure S4 **Temperature optimisation of Hsp12.** ^1^H-^15^N HSQC spectrum of Hsp12 in the presence of 100 mM SDS (A) or in aqueous solution (B) at different temperatures (298, 303, 308, 313, 318, 323 K, Blue -> Red). Increases in temperature are associated with a sharpening of peaks, indicating that HSP12 does not undergo significant unfolding even up to temperatures of 323 K.
(TIF)

Figure S5 **Twenty structures calculated for micelle-bound Hsp12 tiled individually.** Structures were generated using chimera.
(TIF)

Figure S6 **Ramachandran plot.** The percentage of ordered residues in the presence of 100 mM SDS at 45°C was 80.9% in most favoured regions, 18.1% in additionally allowed regions, 0.6% in generously allowed regions and 0.4% in disallowed regions.
(TIF)

Table S1 **Mass spectrometry identifications of proteins induced by dietary restriction and high osmolarity.** Information from the Saccharomyces Genome Database (SGD) is presented along with peptide mass fingerprinting data obtained for each identified protein.
(XLS)

Table S2 **Average RMSD values for Hsp12 helices.** RMSD values calculated from the mean CYANA coordinates for helices I-IV are shown.
(DOC)
