# Supplementary figures and images for: NMR Structure of Hsp12, a Protein Induced by and Required for Dietary Restriction-Induced Lifespan Extension in Yeast
Source: PLoS One. 2012 Jul 27;7(7):e41975. doi: 10.1371/journal.pone.0041975 (PMC3407059; doi:10.1371/journal.pone.0041975)

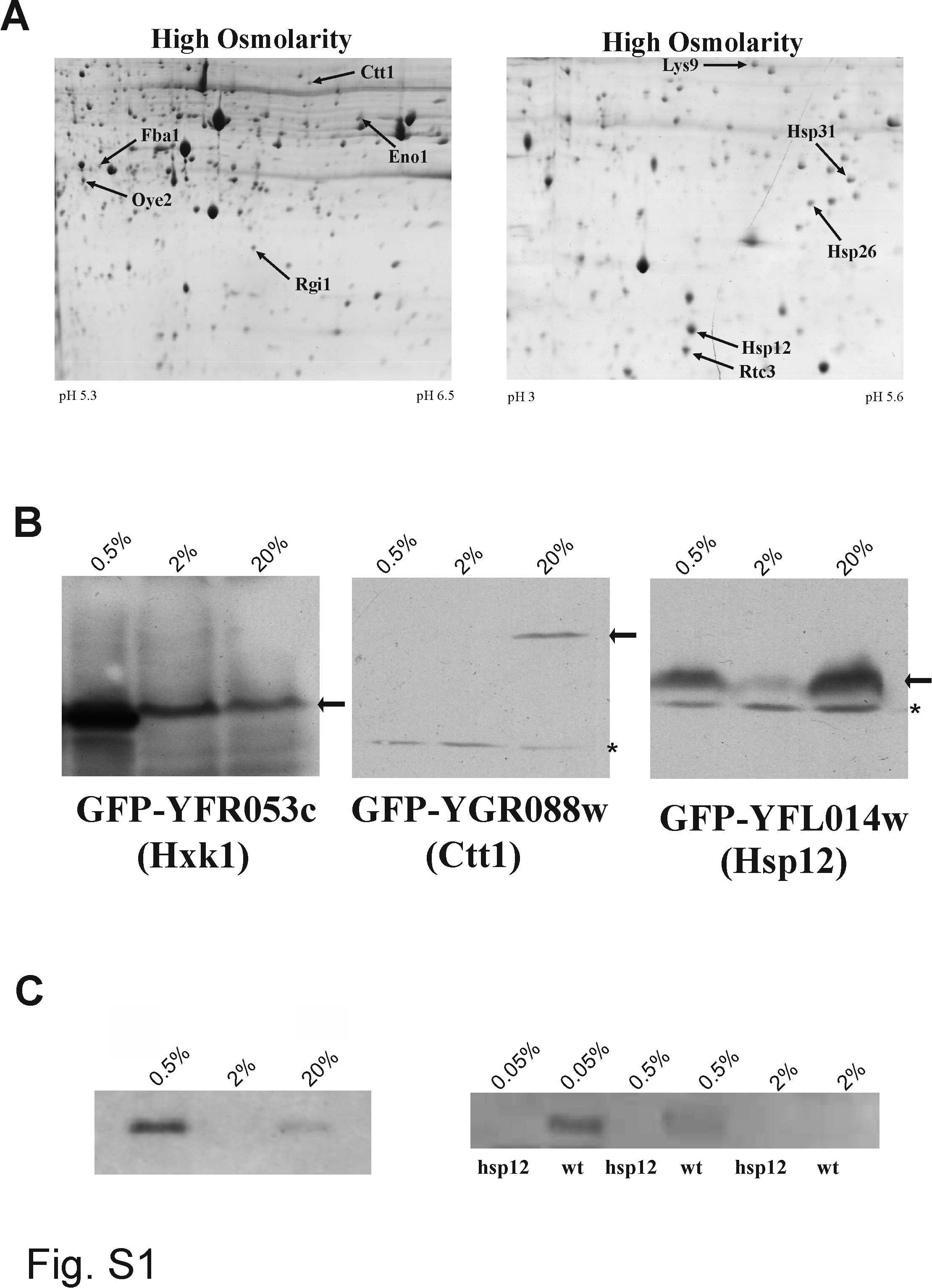

Supplement: Figure S1 — Hsp12 is induced by multiple lifespan-extending interventions. (A) Wild type BY4741 yeast cells were grown in standard (2% glucose) and high osmolarity (20% glucose) conditions before lysis and separation of proteins by 2-D electrophoresis on narrow pH range gels (pH 3.5–6 and 5.3–6.5). Selected spot changes identified by mass spectrometry are indicated by arrows. (B) S288c yeast cells expressing chromosomally GFP-tagged fusions of selected proteins identified as being induced by DR or high osmolarity were grown in 0.5%, 2% and 20% glucose, separated by 1-D SDS-PAGE and western blotted with anti-GFP antiserum. (C) Wild type BY4741 yeast grown in 0.5%, 2% and 20% glucose (left panel); or wild type and isogenic hsp12Δ strains grown in 0.05%, 0.5% and 2% glucose (right panel) were separated by 1-D SDS-PAGE and western blotted with anti-Hsp12 antiserum. (TIF) [file pone.0041975.s001.tif]

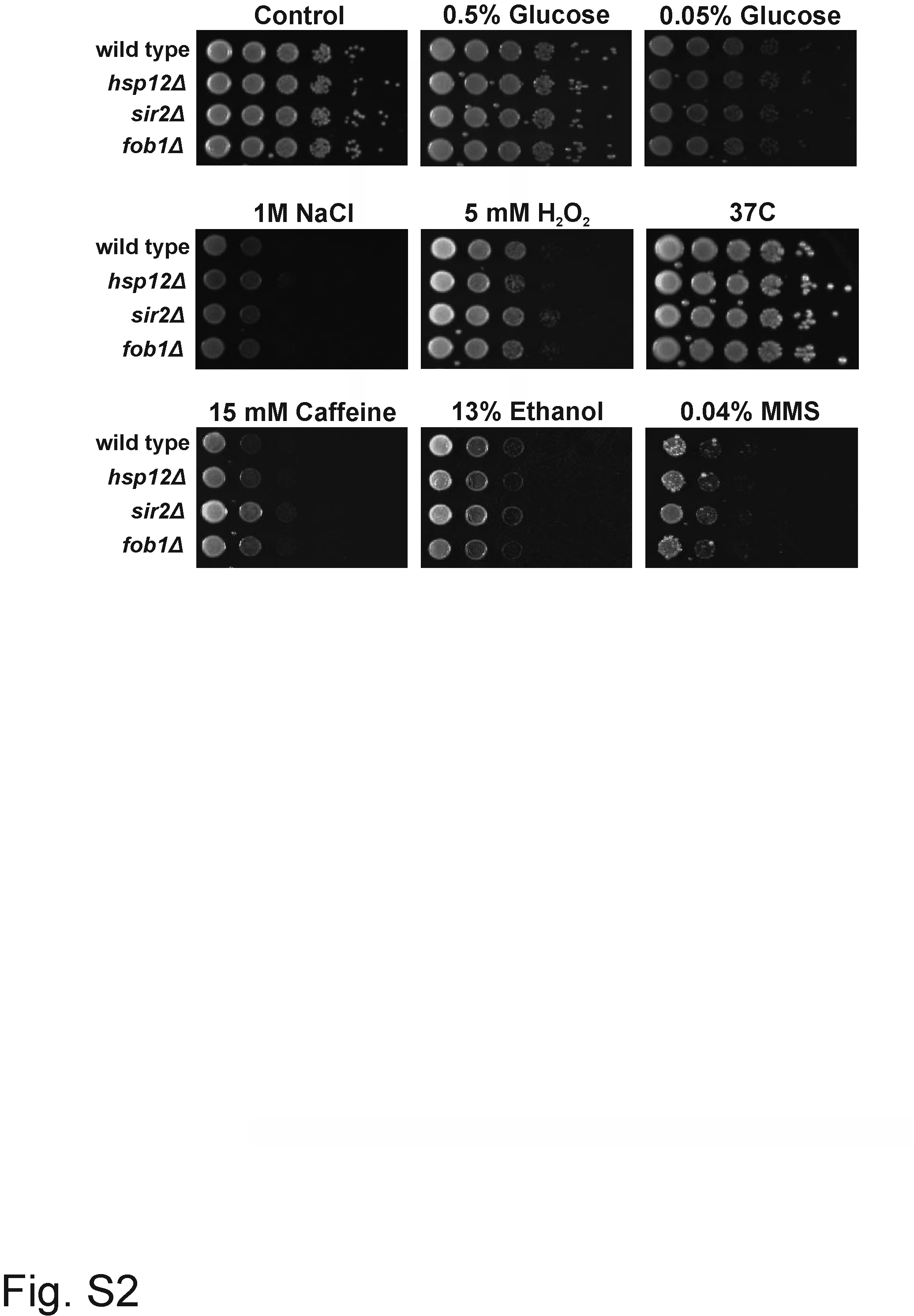

Supplement: Figure S2 — HSP12 is not required for general stress resistance. Overnight cultures of BY4741 wild type and deletion strains were serially diluted and then spotted with a replica plater onto YPD plates containing 2% glucose at 30°C unless indicated otherwise. Plates were incubated at 30°C for 2 to 4 days and then imaged in a BioRad Universal Hood II Imager (BioRad). (TIF) [file pone.0041975.s002.tif]

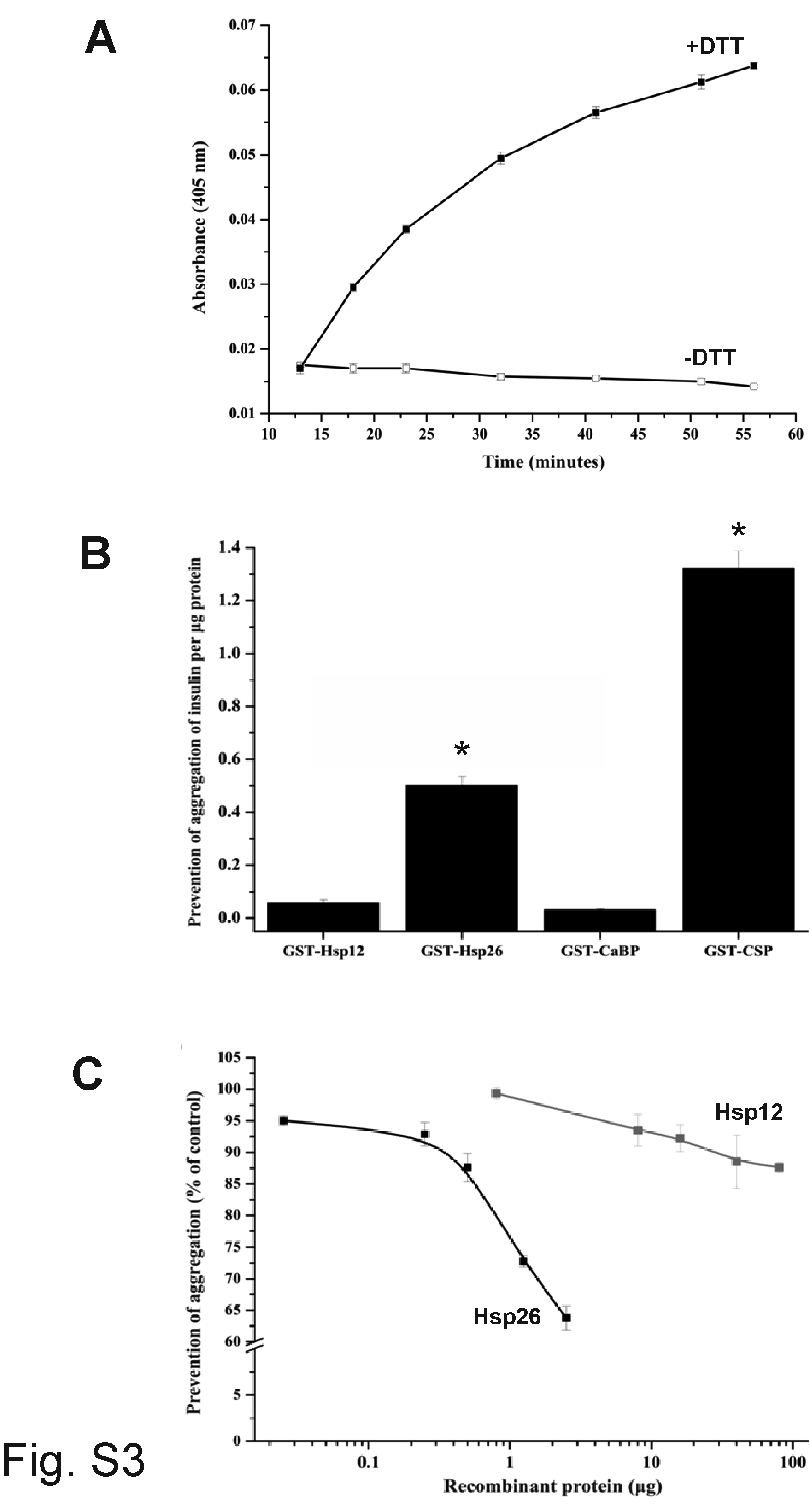

Supplement: Figure S3 — Recombinant Hsp12 has negligible in vitro chaperone activity. (A) 45 µM insulin was supplemented with 1.5 µl water (open squares) or 1 M DTT (black squares) and aggregation over time at room temperature was measured at A405 in a microplate reader. (B) Aggregation assays were performed as above in the presence or absence of the indicated GST-fusion proteins. Anti-aggregation activity is shown as the amount of insulin in µg which is prevented from aggregation by 1 µg of recombinant protein. Data shown are pooled from multiple experiments (n = 7 for GST-Hsp12; n = 8 for GST-Hsp26; n = 4 for GST-CSP; n = 4 for GST-CaBP1s). The difference between GST-Hsp12 and GST-CaBP1s was deemed significant at P<0.05 using a Student’s t-test. (C) Dose-response curves of recombinant GST-Hsp12 and GST-Hsp26. GST-Hsp26 greatly reduces insulin aggregation, whereas GST-Hsp12 has mimimal effect. (TIF) [file pone.0041975.s003.tif]

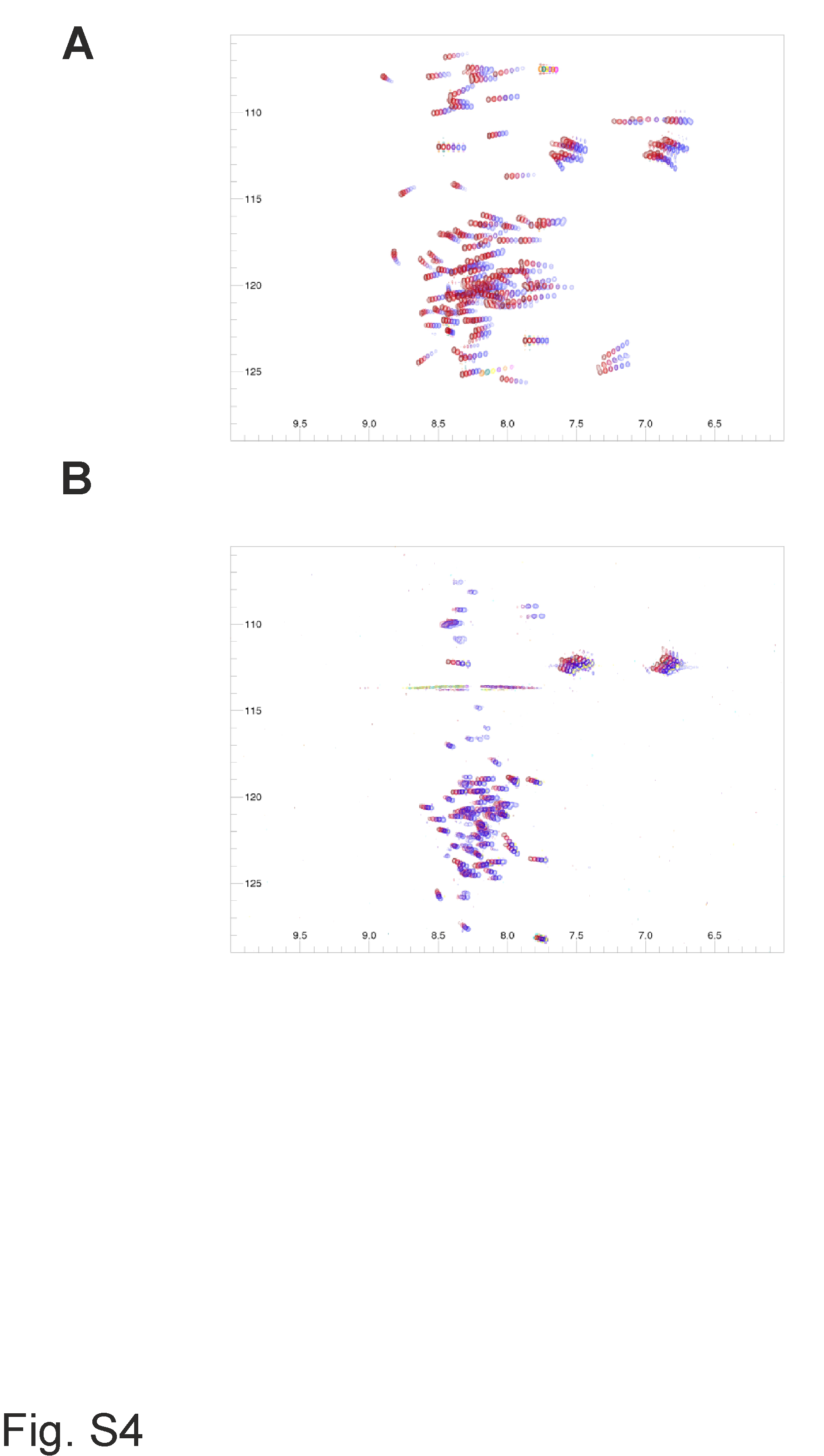

Supplement: Figure S4 — Temperature optimisation of Hsp12. 1H-15N HSQC spectrum of Hsp12 in the presence of 100 mM SDS (A) or in aqueous solution (B) at different temperatures (298, 303, 308, 313, 318, 323 K, Blue -> Red). Increases in temperature are associated with a sharpening of peaks, indicating that HSP12 does not undergo significant unfolding even up to temperatures of 323 K. (TIF) [file pone.0041975.s004.tif]

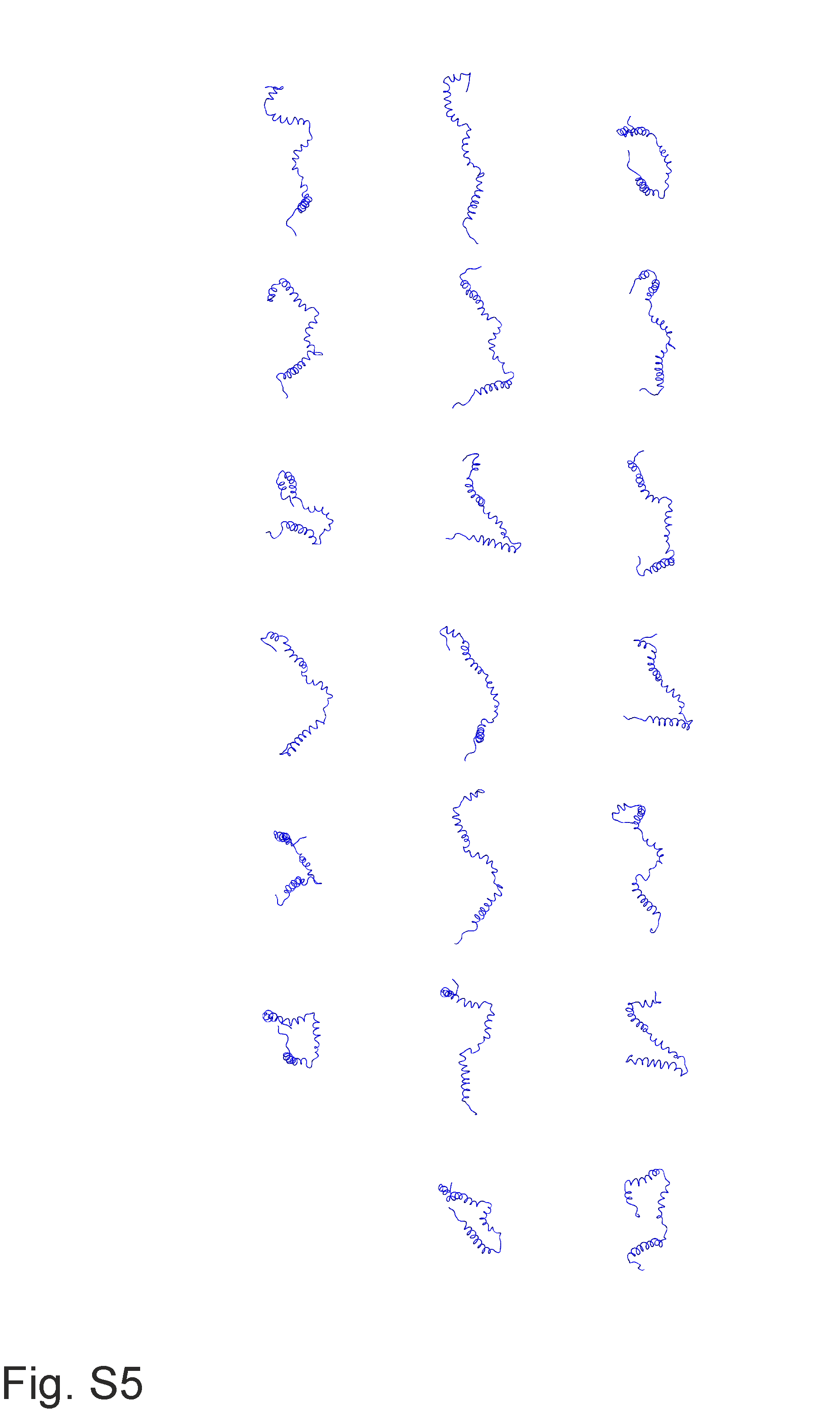

Supplement: Figure S5 — Twenty structures calculated for micelle-bound Hsp12 tiled individually. Structures were generated using chimera. (TIF) [file pone.0041975.s005.tif]

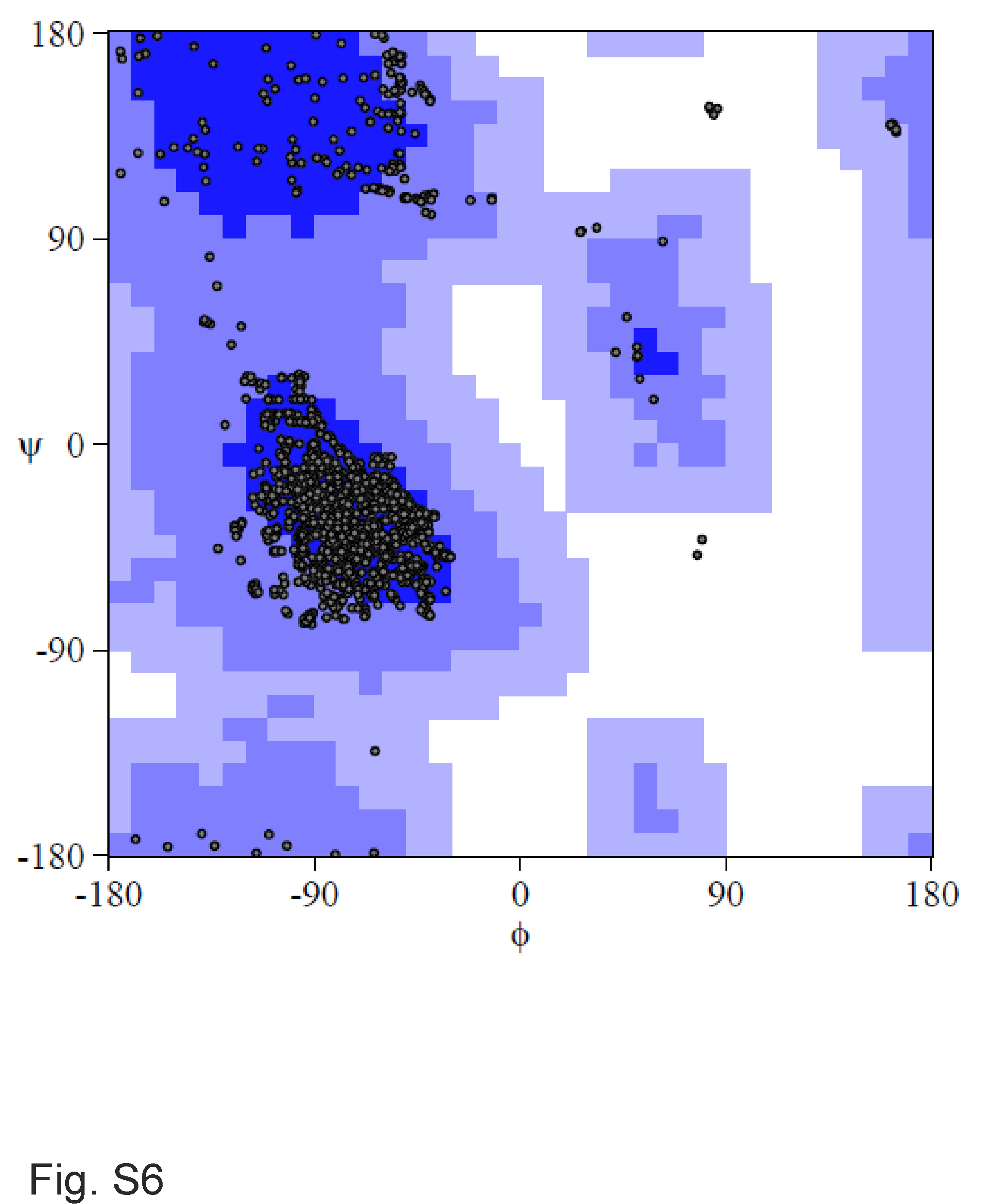

Supplement: Figure S6 — Ramachandran plot. The percentage of ordered residues in the presence of 100 mM SDS at 45°C was 80.9% in most favoured regions, 18.1% in additionally allowed regions, 0.6% in generously allowed regions and 0.4% in disallowed regions. (TIF) [file pone.0041975.s006.tif]
