# Supplementary material for: NMR Structure of Hsp12, a Protein Induced by and Required for Dietary Restriction-Induced Lifespan Extension in Yeast
Source: PLoS One. 2012 Jul 27;7(7):e41975. doi: 10.1371/journal.pone.0041975 (PMC3407059; doi:10.1371/journal.pone.0041975)
Supplement: Table S2 — Average RMSD values for Hsp12 helices. RMSD values calculated from the mean CYANA coordinates for helices I-IV are shown. (DOC) [file pone.0041975.s008.doc]

| **Helix** | **Residues** | **Backbone heavy atoms** | **All heavy atoms** |
| --- | --- | --- | --- |
| Helix I | (PHE9-ALA16) | 0.177 | 1.388 |
| Helix II | (GLN22-ALA41) | 0.364 | 1.242 |
| Helix III | (VAL52-GLY63) | 0.465 | 1.392 |
| Helix IV | (LEU74-GLU94) | 0.497 | 1.330 |
